# The Effects of Pregabalin and the Glial Attenuator Minocycline on the Response to Intradermal Capsaicin in Patients with Unilateral Sciatica

**DOI:** 10.1371/journal.pone.0038525

**Published:** 2012-06-07

**Authors:** Nicole M. Sumracki, Mark R. Hutchinson, Melanie Gentgall, Nancy Briggs, Desmond B. Williams, Paul Rolan

**Affiliations:** 1 Discipline of Pharmacology, School of Medical Sciences, University of Adelaide, Adelaide, Australia; 2 Discipline of Physiology, School of Medical Sciences, University of Adelaide, Adelaide, Australia; 3 Pain and Anaesthesia Research Clinic, University of Adelaide, Royal Adelaide Hospital, Adelaide, Australia; 4 Department of Public Health, University of Adelaide, Royal Adelaide Hospital, Adelaide, Australia; 5 Pharmaceutical Science, School of Pharmacy and Medical Sciences and Sansom Institute for Health Research, University of South Australia, Adelaide, Australia; 6 Discipline of Pharmacology, School of Medical Sciences, University of Adelaide, Adelaide, Australia; Tokyo Metropolitan Institute of Medical Science, Japan

## Abstract

**Background:**

Patients with unilateral sciatica have heightened responses to intradermal capsaicin compared to pain-free volunteers. No studies have investigated whether this pain model can screen for novel anti-neuropathic agents in patients with pre-existing neuropathic pain syndromes.

**Aim:**

This study compared the effects of pregabalin (300 mg) and the tetracycline antibiotic and glial attenuator minocycline (400 mg) on capsaicin-induced spontaneous pain, flare, allodynia and hyperalgesia in patients with unilateral sciatica on both their affected and unaffected leg.

**Methods/Results:**

Eighteen patients with unilateral sciatica completed this randomised, double-blind, placebo-controlled, three-way cross-over study. Participants received a 10 µg dose of capsaicin into the middle section of their calf on both their affected and unaffected leg, separated by an interval of 75 min. Capsaicin-induced spontaneous pain, flare, allodynia and hyperalgesia were recorded pre-injection and at 5, 20, 40, 60 and 90 min post-injection. Minocycline tended to reduce pre-capsaicin injection values of hyperalgesia in the affected leg by 28% (95% CI 0% to 56%). The area under the effect time curves for capsaicin-induced spontaneous pain, flare, allodynia and hyperalgesia were not affected by either treatment compared to placebo. Significant limb differences were observed for flare (AUC) (−38% in affected leg, 95% CI for difference −19% to −52%). Both hand dominance and sex were significant covariates of response to capsaicin.

**Conclusions:**

It cannot be concluded that minocycline is unsuitable for further evaluation as an anti-neuropathic pain drug as pregabalin, our positive control, failed to reduce capsaicin-induced neuropathic pain. However, the anti-hyperalgesic effect of minocycline observed pre-capsaicin injection is promising pilot information to support ongoing research into glial-mediated treatments for neuropathic pain. The differences in flare response between limbs may represent a useful biomarker to further investigate neuropathic pain. Inclusion of a positive control is imperative for the assessment of novel therapies for neuropathic pain.

## Introduction

The management of chronic neuropathic pain is a major unmet medical need [1] with few new classes of drugs reaching clinical practice and none of which are disease modifying. Recently, the potential role of glial activation in initiating and promoting the development of chronic neuropathic pain has been convincingly demonstrated in animals [2], [3], [4]. However, there is no access to the central nervous system and no validated imaging tools for assessing glial activation in humans. Hence, the determination of the role of glial activation in humans is difficult, with functional assessment being the only current feasible method that can be used. Although there have been small trials of potential glial inhibitors in clinical trials [5], [6], [7], [8], [9], [10], [11], [12], [13], [14], [15], no adequately powered trial has been reported. Trials in chronic pain are notoriously difficult in which to elicit a drug signal due to large and variable placebo response, regression to the mean and participant withdrawal [16]. An alternative method for seeking such a signal is to perform a carefully standardised neuropathic-like stimulus (pain model) in patients with chronic pain. It has been suggested that a chronic state of glial activation may exist in patients with neuropathic pain; hence novel disease-modifying therapies need to be investigated in the diseased patient (i.e. patients with neuropathic pain) as investigation of such disease modifying therapies in healthy volunteers is likely to result in a false negative response due to the likely quiescent state of glia in healthy volunteers.

We report such a trial where intradermal capsaicin, intended to produce a transiently heightened neuropathic-like state, has been applied to the limbs of patients with unilateral sciatica, a condition of mixed basis [17] but with some neuropathic element in many patients. In the current study however, we attempted to determine whether the glial inhibitor and tetracycline antibiotic, minocycline, could reduce the response to intradermal capsaicin. Previously, numerous rodent studies have demonstrated that minocycline attenuates glial activation and possesses both neuroprotective and anti-inflammatory properties independent of its antibiotic activity [18]. Furthermore, the use of minocycline as a potential glial inhibitor in humans is justified due to its well known safety profile [19]; its ability to readily penetrate the blood-brain brain barrier [20], thereby gaining access to the central nervous system; and its ability to safely reach therapeutic levels in humans. However, minocycline only appears to be effective in reducing neuropathic pain in rodents if administered pre-emptively [21] and hence might not reduce pre-existing neuropathic pain, but might attenuate the response to a new neuropathic-like stimulus. We have previously demonstrated that intradermal capsaicin produces a transiently heightened neuropathic-like state in both healthy volunteers [22] and in patients with unilateral sciatica [23] with a dose-response relationship. Hence intradermal capsaicin was selected to safely provide such a stimulus. Given the difficulty in interpreting a negative response, we have attempted to incorporate a positive control for the response to capsaicin. We selected pregabalin as the positive control due to its modest, yet non-statistically significant reduction of capsaicin-induced secondary hyperalgesia observed by Wang and colleagues [24] in healthy male volunteers.

We hypothesised that pregabalin would reduce capsaicin-induced spontaneous pain, flare, allodynia and hyperalgesia as evidence of general anti-neuropathic effects and that minocycline would also attenuate the response to capsaicin by attenuation of the glial contributions to the new neuropathic-like pain stimulus that the capsaicin challenge produces.

## Materials and Methods

### Participant Selection

Sciatica was diagnosed on clinical grounds by the presence of pain in the L5/S1 dermatomal distribution accompanied by dysaethesia of a shocking or burning quality of pain. Participants were to have negligible pain symptoms in their contralateral leg. The first and last patients were randomised on the 29^th^ of June 2009 and the 27^th^ of July 2009 respectively.

### Inclusion and Exclusion Criteria

Key inclusion criteria were: being between 18 and 65 years old inclusive; having both lower limbs present; and having suffered from unilateral sciatica for a minimum of 3 months, with this being their dominant pain problem. Additionally, women of childbearing potential using hormonal contraception were only eligible if they agreed to additional barrier contraception until their first normal period after the study, due to contraindications of minocycline and must have had a negative pregnancy test prior to each testing session. Exclusion criteria included: known intolerance to capsaicin, pregabalin or minocycline; being pregnant or breastfeeding; scarring or tattoos on either leg at the planned site of injection; deep skin pigmentation which would have precluded the flare assessment; and recent use of opioids (e.g. morphine use within 1 week) or pain modifying drugs (e.g. tricyclic antidpressants, gabapentin or pregabalin use within 1 month). Participants on paracetamol or non-steroidal anti-inflammatory drugs (NSAIDs) were required to withhold medication for 24 h or 5 half lives of the medication, whichever was longer, prior to each study day to exclude any effect on the capsaicin response. Participants were also excluded if their creatinine clearance as estimated by Cockcroft-Gault was less than 60 mL/min.

### Ethics

Ethics approval was obtained from the Royal Adelaide Hospital Research Ethics Committee. Signed consent was obtained from each participant prior to enrolment into the study and participants were financially compensated for their time and inconvenience.

### Capsaicin Preparation

Capsaicin in 38% hydroxypropyl-β-Cyclodextrin (β-CD) was prepared and dispensed as described previously [22]. A 10 µg dose was selected based on previous work from our laboratory [23]. For each injection, 10 µL of solution was drawn into a 0.3 mL sterile insulin syringe (BD Ultra-Fine II) by the Royal Adelaide Hospital Department of Pharmacy.

### Familiarisation

As part of the screening session, participants were familiarised to the four outcome measures of spontaneous pain, area of flare, allodynia and hyperalgesia, described below, in response to a single intradermal capsaicin injection of 10 µg in 10 µL 38% hydroxypropyl-β-CD. All outcome measures were tested before and 5 min post-injection.

### Assessment Procedures

This randomised, double-blinded, placebo-controlled, double-dummy, three-way cross over study was conducted over 3×5 h sessions, with a one week washout in between each session. The three study treatments were single oral doses of minocycline, pregabalin and placebo. Order of treatment allocation was randomised using a Latin square design to control for any potential carry-over effect between study days. On each dosing occasion, participants received either an oral dose of (1) minocycline (400 mg) at −2 h and placebo pregabalin at −1 h; or (2) placebo minocycline at −2 h and pregabalin (300 mg) at −1 h; or (3) placebo minocycline at −2 h and placebo pregabalin at −1 h before the first intradermal capsaicin injection, according to a double-dummy design. The time points for study treatment administration were chosen based on the pharmacokinetic profiles of both minocycline and pregabalin, to ensure that both capsaicin injections would be administered as equally as possible to either side of minocycline or pregabalin’s plasma peak concentration. An equal number of capsules of the active and placebo treatments were administered orally to each participant to avoid potential un-blinding. Therefore, minocycline was administered as 4×100 mg capsules; pregabalin as 4×75 mg capsules and placebo as 4×starch capsules. Due to dissimilarities between the capsules, a member of staff (who were otherwise not involved in any of the assessments) administered the different treatments and the participants were blindfolded throughout treatment administration to avoid treatment allocation un-blinding. Throughout the assessments, participants lay prone on a bed, and the skin temperature of the test site was fixed at 34–36°C, using a 250 W infrared heat lamp positioned approximately 50 cm from the back of the calf, as this has been previously shown to reduce the variability of the intradermal capsaicin pain model in the volar forearm of pain free volunteers [25]. The temperature was monitored using a thermode on the adjacent skin. Participants received two injections of intradermal capsaicin per dosing occasion, with each injection separated by an interval of 75 min. The injections were administered to the middle section of the calf on both their affected and unaffected leg, with the order of injections according to a random code. Spontaneous pain, area of flare, allodynia and hyperalgesia (in that order) were measured prior to and at 5, 20, 40, 60 and 90 min post injection. All assessments were measured and recorded by the same assessor to maintain consistency between measurements. Collectively, all four assessments took about 3 min to complete.

1. **Spontaneous pain.** Capsaicin-induced spontaneous pain was assessed using a 100 mm visual analogue scale (VAS). Participants were required to mark the severity of their pain from a score of 0, indicating ‘no pain’ to 100, signifying ‘the worst pain imaginable’. The length (mm) was recorded with a ruler.
2. **Flare.** Capsaicin-induced area of flare (cm^2^) was assessed by tracing the visually identified area of reddened skin directly onto an overlaying transparent acetate sheet, using a soft-tipped pen [23]. The area was then calculated using digital planimetry.
3. **Allodynia.** The average radius (cm) of capsaicin-induced allodynia was assessed using a foam paint brush (Foam brush 2*ROYMAC, Australia), gently stroked across the skin along 8 compass directions as previously described [23]. The average sum of each radii between the marked dot and the injection site was later determined. This has been found to be more appropriate than measuring an area of allodynia or hyperalgesia, as not all assessments result in eight points of response [23].
4. **Hyperalgesia.** The average radius (cm) of capsaicin-induced hyperalgesia was assessed using a calibrated von Frey hair, a plastic rod that bends at a defined pressure (SENSELab monofilaments, Somedic, Horby, Sweden). The von Frey hair was applied along 8 compass directions as described above for allodynia. Participants were instructed to say ‘yes’ if they noticed an ‘increase in pain’ or a ‘change in sensation’, and the average of these points was then recorded and measured as described for allodynia. The von Frey hair selected for each participant was the highest von Frey hair that produced discomfort but no pain prior to intradermal capsaicin at the screening session. This hair was selected by applying each von Frey hair (from lowest to highest) three times to the middle section of the calf on the participants affected leg until the participant reported a painful response. The von Frey hair below the one that elicited a painful response was then selected, and was used for the entire duration of the study for that particular participant.

### Statistics

A power calculation was performed based on a previous study investigating intradermal capsaicin and pregabalin in healthy volunteers [24] to detect a 25% difference between treatments with an alpha level of 0.05. This requried between 11 and 18 participants to ensure 80% power in detecting a change for a two-tailed test. Given the uncertainty of the effect size of minocycline and pregabalin in patients with unilateral sciatica, and the exploratory nature of this study, a sample size of 18 was selected. The primary endpoint was to compare the effects of minocycline and pregabalin with placebo on capsaicin induced spontaneous pain (mm), area of flare (cm^2^), allodynia (average radius, cm) and hyperalgesia (average radius, cm) in patients with unilateral sciatica, on both their affected and unaffected leg. A linear mixed effects model was fitted to each of the primary endpoints predicted by order of injection (affected or unaffected leg); order of treatment and time, and their interactions. The model assesses the interaction between variables and subsequently is used to estimate point estimates and the 95% confidence intervals for each variable and for differences between variables. Main effects of sex; hand dominance and affected leg status were included as covariates in this model. The effect of intradermal capsaicin on spontaneous pain, flare, allodynia and hyperaglesia (placebo data only) was analysed using a one-way repeated measures (RM) ANOVA. Holm’s Stepdown Bonferroni adjustment was performed to adjust for multiple comparisons. Statistical analysis was performed using the SAS 9.1 Program, SAS Institute Inc., Cary, NC, USA and Prism software version 5 (GraphPad Software, San Diego, CA, USA). A *P* value of less than 0.05 was required for statistical significance. All *P* values are expressed as unadjusted values unless otherwise stated.

## Results

### Subjects

18 participants with unilateral sciatica (13 males and 5 females, mean age 49.4±11.4 years) completed this study. An additional participant withdrew after the first study day, due to unknown reasons. Unblinding revealed that this participant received placebo treatment. Their placebo data were included in the analyses. The duration of disease ranged from 10 months to 24 years, with a mean of 7.7 years (95% CI : 3.9 to 11.5 years). All participants were Caucasian. Demographics and concomitant medications are listed in Table 1.

**Table 1:** Patient Demographics.

Sex (M/F)		13/5
Age (years, mean ± SD)		49.4±11.4
Race (%)	*Caucasian*	100
Duration of Disease(years ± SD)		7.7±7.7
Concominant Medications(%)	*Paracetamol*	48
	*Paracetamol + Codeine*	5
	*NSAIDs*	5
	*NSAIDs + Codeine*	21
	*Diazepam*	16

NSAIDs: Nonsteroidal anti-inflammatory drugs.

### Tolerability and Safety

Capsaicin doses were well tolerated by all participants, with no adverse effects reported or observed, other than the expected local responses. Minocycline doses were well tolerated by most participants, with only two participants experiencing adverse effects which included dizziness; difficulty focussing and headache. Fourteen participants experienced adverse effects such as dizziness, nausea and tiredness following pregabalin treatment. One participant reported tiredness following placebo. Most adverse effects, such as dizziness and nausea, were not reported or observed until the end of the study day, when participants sat upright, therefore not un-blinding the investigator throughout the assessments.

### VAS Score

**Figure 1:**
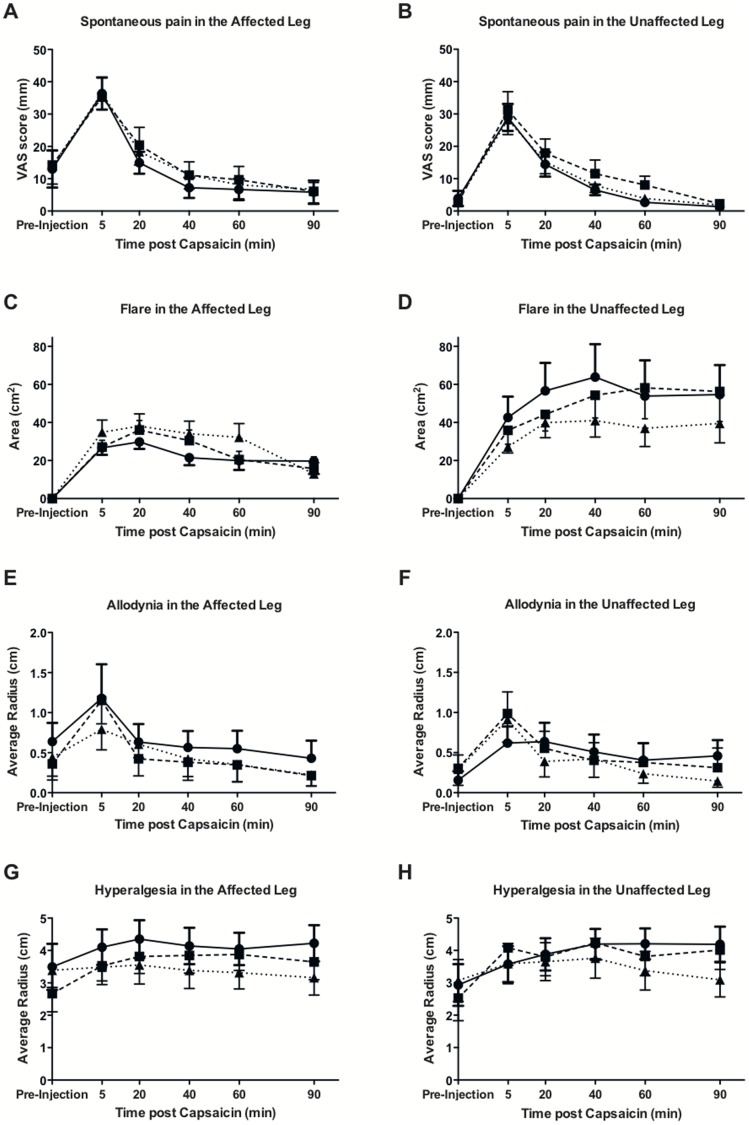
Capsaicin-induced spontaneous pain, flare, hyperalgesia and allodynia. Time course of capsaicin-induced spontaneous pain, flare, allodynia and hyperalgesia responses following placebo (•), single oral dose minocycline (400 mg ▪) and single oral dose pregabalin (300 mg ▴) in patients with unilateral sciatica in their affected (A, C, E, G) and unaffected (B, D, F, H) leg. Data presented as mean ± SEM.

Effect-time profiles are shown in Figure 1 panels A (affected leg) and B (unaffected leg). Baseline values of pain in the placebo group were 13 mm (95% CI 1 mm to 25 mm) in the affected leg, indicating a low baseline level of symptoms, and 4 mm (95% CI −0.1 mm to 9 mm) in the unaffected, confirming the largely unilateral nature of symptoms. The effect-time profiles for the VAS scores following capsaicin in both the affected leg and unaffected leg were similar, with the peak response occurring at t = 5 min, followed by a gradual decline over time. The AUC (see table 2 for all AUC values) of VAS scores was 28% (95% CI 8% to 43%) lower in the unaffected leg compared to the affected leg. However, once adjusting for multiple comparisons, statistical significance was lost (p>0.05). Neither pregabalin nor minocycline reduced the AUC of VAS scores in either the affected or unaffected leg (p>0.05). A one-way RM ANOVA demonstrated that the 10 µg dose of intradermal capsaicin used caused a significant increase in pain from baseline at t = 5 min (23 mm increase, 95% CI for increase 13 mm to 34 mm) in the affected leg [F (5, 18) = 16.61, p<0.0001] and at t = 5 min (25 mm increase, 95% CI for increase 17 mm to 34 mm) and t = 20 min (11 mm increase, 95% CI for increase 2 mm to 19 mm) in the unaffected leg [F (5, 18) = 21.23, p<0.0001] when placebo was administered, demonstrating intradermal capsaicin’s ability to induce a level of spontaneous pain that is generally considered at the lower end of clinically significant pain (∼40 mm).

**Table 2:** AUC of capsaicin-induced spontaneous pain, flare, allodynia and hyperalgesia in patients with unilateral sciatica in their affected and unaffected leg.

Outcome Measured	Treatment	Mean AUC (95% CI)	
		Affected Leg	Unaffected Leg
Spontaneous pain	Placebo	656.5 (386.9, 1114.1)	489.7 (288.6, 831.0)
	Pregabalin	752.5 (422.9, 1338.8)	485.6 (272.9, 864.0)
	Minocycline	750.8 (420.3, 1341.3)	592.0 (331.3, 1057.4)
Flare	Placebo	1475.2 (985.0, 2210.0)	3176.2 (2120.6, 4757.4)
	Pregabalin	1936.3 (1244.2, 3013.2)	2286.7 (1469.4, 3558.6)
	Minocycline	1673.6 (1049.0, 2671.0)	2766.1 (1733.1, 4414.7)
Allodynia	Placebo	28.9 (12.3, 68.1)	17.2 (7.4, 40.7)
	Pregabalin	23.9 (11.9, 48.0)	20.5 (10.2, 41.1)
	Minocycline	15.5 (7.0, 34.3)	16.2 (7.3, 35.9)
Hyperalgesia	Placebo	373.2 (282.3, 464.1)	363.0 (272.0, 453.9)
	Pregabalin	303.5 (209.8, 397.1)	306.9 (213.2, 400.5)
	Minocycline	337.3 (241.4, 433.1)	354.0 (258.2, 449.8)

AUC: Area under the curve.

### Area of Flare

Effect time profiles are shown in Figure 1, panels C (affected leg) and D (unaffected leg). Baseline flare values were zero in the affected and unaffected leg, as expected. The time profiles for area of flare differed in the affected and unaffected leg. In the affected leg, the peak response occurred at t = 20 min for all treatments, followed by a decline over the 90 min session. In the unaffected leg, the response appeared to increase and stay steady over the 90 min session, with the peak response at t = 40 min for placebo and pregabalin, and at t = 60 min for minocycline. Neither pregabalin nor minocycline had an effect on the areas of flare in either the affected or the unaffected leg (p>0.05). The AUC of flare was significantly lower in the affected leg by 38% (95% CI 19% to 52%, p = 0.024) (adjusted p value) compared to the unaffected leg. A one-way RM ANOVA demonstrated that the 10 µg dose of intradermal capsaicin caused a significant increase in mean (95% CI) flare from baseline at t = 5 min : 26.7 cm^2^ (14.7–38.5 cm^2^), t = 20 min : 29.8 cm^2^ (17.9–41.7 cm^2^), t = 40 min : 21.5 cm^2^ (9.6–33.4 cm^2^), t = 60 min : 20.0 cm^2^ (8.1–31.6 cm^2^) and t = 90 min : 19.7 cm^2^ (7.8–31.6 cm^2^) in the affected leg [F (5, 18) = 10.59, p<0.0001] and at t = 5 min : 42.6 cm^2^ (12.8–72.4 cm^2^), t = 20 min : 56.7 cm^2^ (26.9–86.5 cm^2^), t = 40 min : 63.9 cm^2^ (34.1–93.6 cm^2^), t = 60 min : 53.9 cm^2^ (24.1–83.7 cm^2^) and t = 90 min : 54.6 cm^2^ (24.9–83.4 cm^2^) in the unaffected leg [F (5, 18) = 8.41, p<0.0001] when placebo was administered. Significant differences were observed between the areas of flare in the affected and unaffected leg at t = 40 min (p = 0.0078), t = 60 min (p = 0.038) and t = 90 min (p = 0.0068) for placebo, and at t = 90 min for both minocycline (p = 0.0059) and pregabalin (p = 0.021), demonstrating an increasing area of flare in the unaffected leg over the 90 min session.

### Allodynia

Effect time profiles are shown in Figure 1 panels E (affected leg) and F (unaffected leg). Positive allodynia baseline values were observed in both the affected (average radius 0.6 cm, 95% CI 0.2 cm to 1.1 cm) and unaffected leg (average radius 0.2 cm, 95% CI −0.08 cm to 0.4 cm) in the placebo group. Mean allodynia responses appeared to resolve to pre-capsaicin injection values at a faster rate in the affected leg compared to the unaffected. There was no significant difference between the means over the 90 min session between the affected and the unaffected leg. Neither pregabalin nor minocycline reduced the AUC of allodynia in either the affected or the unaffected leg (p>0.05). A one-way RM ANOVA demonstrated that the 10 µg dose of intradermal capsaicin used caused a significant, although very small, increase in allodynia from baseline at t = 5 (0.5 cm increase, 95% CI for increase 0 cm to 1.1 cm) in the affected leg [F (5, 18) = 3.31, p = 0.0087] and at t = 5 min (0.5 cm increase, 95% CI for increase 0 cm to 0.9 cm) and t = 20 min (0.5 cm increase, 95% CI for increase 0 cm to 0.9 cm) in the unaffected leg [F (5, 18) = 2.27, p = 0.054] when placebo was administered.

### Hyperalgesia

Effect time profiles are shown in Figure 1 panels G (affected leg) and H (unaffected leg). Positive hyperalgesia baseline values were observed in both the affected and unaffected leg, with slightly higher values in the affected leg. Minocycline reduced pre-capsaicin injection values of hyperalgesia (average radius, cm) in the affected leg by approximately 28% compared to placebo, with borderline significance (95% CI 0% to 56%, p = 0.053). However, once adjusting for multiple comparisons, statistical significance was lost (p = 0.91). There was no significant difference between the means over the 90 min session between the affected and unaffected leg. Neither pregabalin nor minocycline reduced the AUC of hyperalgesia in either the affected or the unaffected leg (p>0.05). A one-way RM ANOVA demonstrated that the 10 µg dose of intradermal capsaicin used was insufficient to produce increased hyperalgesia responses from baseline in the affected leg when placebo was administered [F (5, 18) = 1.12, p = 0.31]. However, the 10 µg dose of intradermal capsaicin used caused a significant, although very small, increase in hyperalgesia from baseline in the unaffected leg at t = 20 min (0.9 cm increase, 95% CI for increase 0.1 cm to 1.8 cm), t = 40 min (1.3 cm increase, 95% CI for increase 0.4 cm to 2.1 cm), t = 60 min (1.3 cm increase, 95% CI for increase 0.4 cm to 2.1 cm) and t = 90 min (1.3 cm increase, 95% CI for increase 0.4 cm to 2.1 cm) when placebo was administered [F (5, 18) = 5.01, p = 0.0004].

## Discussion

The scarcity of experimental pain studies in patients with chronic pain makes the interpretation of results generated by studies investigating the efficacy of analgesics in pain-free participants difficult in patients with chronic pain, as it is unknown whether the mechanistic processing of experimental pain differs between patients with chronic pain and pain-free participants. If indeed the mechanistic processing of experimental pain differs between patients with chronic pain and pain-free participants, the response to challenge agents, such as capsaicin, and the response to both positive and negative controls will not necessarily be similar in patients with chronic pain and pain-free participants. Therefore, further investigation of experimental pain is required in patients with chronic pain. Although we report a negative result, these findings are important for others working in this research area when considering experimental design factors.

In this study we have not detected anti-neuropathic effects of either the positive control pregabalin or the experimental drug minocycline. The study design was based on that of Wang *et al*. [24], who showed a non-statistically significant reduction in hyperalgesia to 100 µg of capsaicin in healthy volunteers following pregabalin. In our previous study (Aykanat *et al*., [23]), comparing the responses to intradermal capsaicin between healthy volunteers and patients with unilateral sciatica, we saw a considerably enhanced response to all variables in the patients and hence used a lower dose of capsaicin (10 µg) than that used in the study of Wang *et al.,* [24], as we were concerned that higher doses might not be tolerated. Following 10 µg of intradermal capsaicin, we observed increased responses to the variables spontaneous pain, flare and allodynia in both the affected and unaffected limb and hyperalgesia in the unaffected limb compared to pre-capsaicin injection responses, whereas hyperalgesia in the affected limb remained unaltered post-capsaicin injection compared to pre-capsaicin injection. Given that there was no increase in hyperalgesia in the affected limb, and only small increases in hyperalgesia in the unaffected limb following capsaicin, it is not surprising that we saw no effect of the positive control, as a reduction in post-capsaicin hyperalgesia was the primary outcome. Given the capsaicin was very well tolerated in both this study and in our previous study [23], and that it is essential to produce a robust hyperalgesia profile, we propose to use a higher dose of capsaicin in future studies.

Of particular interest is the difference observed in flare responses between the affected and unaffected limb of patients with unilateral sciatica. Similar to Aykanat *et al.,* [23], flare responses were lower in the affected leg compared to the unaffected leg of unilateral sciatica patients. Lower capsaicin-induced flare responses have also been observed in the affected regions of lower lumbar pain [26] and post-herpetic neuralgia (PHN) patients [26], [27], [28] when compared to pain patients flare responses in an unaffected body site. Morris *et al.,* [27] demonstrated that PHN patients who experience allodynia have lower capsaicin-induced flare responses compared to PHN patients who do not experience allodynia and healthy volunteers. However, no correlation between the amount of allodynia experienced and capsaicin-induced flare was demonstrated in this study. Flare has previously been attributed to an axon reflex of C-fibres. Consequently, neuronal loss may result in reduced flare responses. Although the participants in this study did not present clinical evidence of sensory loss, it has previously been demonstrated that patients with type 2 diabetes without clinical signs of neuropathy had significantly reduced flare responses compared to healthy volunteers when flare was induced by heating the skin to 44°C [29]. Accordingly, our results support the abovementioned previous findings of reduced flare responses in the painfully affected body region; and give further support to a neuropathic component in sciatica. Alternatively the reduced flare observed in the affected leg may be due to increased inhibitory control. If true, the capsaicin-induced flare response may represent a biomarker of descending inhibitory control.

Only three studies have previously investigated the response to intradermal capsaicin in patients with pain; with these being in vulvodynia-afflicted women [30], patients with rheumatoid arthritis [31] and patients with unilateral sciatica [23]. Shenker *et al.,* [31] investigated bilateral capsaicin-induced hyperalgesia and allodynia in response to intradermal capsaicin administered unilaterally, thus did not compare capsaicin-induced responses between patients with rheumatoid arthritis and pain-free controls in the injected capsaicin limb [31]. However, the studies in vulvodynia-afflicted women [30] and patients with unilateral sciatica [23] demonstrated significantly heightened capsaicin-induced responses in patients with pain compared to pain-free controls, supporting the feasibility of investigating this neuropathic pain model in patients with neuropathic pain. To our knowledge the current study is the first study investigating whether the intradermal capsaicin pain model can screen for novel anti-neuropathic agents in patients with pre-existing neuropathic pain.

Although not significant once adjusting the *P*-value, the 28% (95% CI 0% to 56%) reduction of hyperalgesia in the affected leg prior to intradermal capsaicin by single oral dose minocycline is a novel finding that glial attenuation may be anti-hyperalgesic in humans. Similar results have been demonstrated in animal studies, where minocycline administration post-spinal cord injury for five consecutive days significantly increased mechanical nociceptive thresholds in male Sprague-Dawley rats compared to vehicle treated rats [32]. However, the absence of pre-drug administration assessments makes interpretation of this reduced pre-capsaicin baseline following minocycline difficult.

Pregabalin did not reduce capsaicin-induced spontaneous pain. Although there was a clear capsaicin effect, the main peak effect was below the 40 mm value generally accepted as the minimum for clinically relevant pain [33]. This is further argument to use a higher dose of capsaicin in future studies in order to produce clinically relevant pain, which may be attenuated by the experimental drug. The use of pregabalin as our positive control was warranted, compared to an opioid analgesic, as pregabalin is amongst the recommended first-line treatments for neuropathic pain, as recommended by the Neuropathic Pain Special Interest Group of the International Association for the Study of Pain [34], the Canadian Pain Society [35] and the European Federation of Neurological Studies [36]; whereas opioids are generally considered as a second-line or even third-line treatment for neuropathic pain. Consequently, we wished to incorporate a recommended first-line treatment for neuropathic pain as our positive control.

Pregabalin, a licenced treatment for neuropathic pain, was incorporated into this study as a positive control. As oral pregabalin failed to significantly reduce capsaicin-induced neuropathic pain in patients with unilateral sciatica, it cannot be concluded that minocycline is unsuitable for further evaluation as an anti-neuropathic pain drug. Therefore, the anti-hyperalgesic effect observed following single oral dose administration of minocycline is promising pilot information to support ongoing research into glial mediated treatments for neuropathic pain. The findings from this study demonstrate the importance of incorporating a positive control when assessing the efficacy of novel therapies, especially when the effect size of the experimental stimulus has not been validated in the patient population being tested. Therefore, future studies utilising the capsaicin-induced neuropathic pain model in a chronic pain patient population should ensure that the capsaicin dose selected is an adequate dose to produce a reliable area of secondary hyperalgesia and allodynia, whilst maintaining a stimulus intensity that can be tolerated satisfactorily. Additionally, capsaicin-induced flare responses in chronic pain patients may provide a simple, objective biomarker to further investigate neuropathic pain.

### Limitations

The key limitation of this study is the unexpectedly low hyperalgesia produced by capsaicin. This made the detection of a drug-induced reduction, whether to the positive control or experimental drug, difficult. Further studies should use a higher dose of capsaicin.
